# A Micro-Raman Study of Live, Single Red Blood Cells (RBCs) Treated with AgNO_3_ Nanoparticles

**DOI:** 10.1371/journal.pone.0103493

**Published:** 2014-07-24

**Authors:** Aseefhali Bankapur, Surekha Barkur, Santhosh Chidangil, Deepak Mathur

**Affiliations:** 1 Centre for Atomic and Molecular Physics, Manipal University, Manipal, India; 2 Tata Institute of Fundamental Research, Mumbai, India; Wayne State University School of Medicine, United States of America

## Abstract

Silver nanoparticles (Ag NPs) are known to exhibit broad antimicrobial activity. However, such activity continues to raise concerns in the context of the interaction of such NPs with biomolecules. In a physiological environment NPs interact with individual biological cells either by penetrating through the cell membrane or by adhering to the membrane. We have explored the interaction of Ag NPs with single optically-trapped, live erythrocytes (red blood cells, RBCs) using Raman Tweezers spectroscopy. Our experiments reveal that Ag NPs induce modifications within an RBC that appear to be irreversible. In particular we are able to identify that the heme conformation in an RBC transforms from the usual R-state (oxy-state) to the T-state (deoxy-state). We rationalize our observations by proposing a model for the nanoparticle cytotoxicity pathway when the NP size is larger than the membrane pore size. We propose that the interaction of Ag NPs with the cell surface induces damage brought about by alteration of intracellular pH caused by the blockage of the cell membrane transport.

## Introduction

The well-established antimicrobial activity of silver nanoparticles (Ag NPs) continues to be of importance in a plethora of industries, such as those concerned with antimicrobial coatings, food storage, water purification, textiles, keyboards, wound dressings, apparel, footwear, paints, appliances, cosmetics, and plastics, and increasingly, also in biomedical devices. One of the most relevant facets of Ag NPs is the broad spectrum of their antimicrobial activities *vis-à-vis* bacteria, fungi, and viruses [1]. Silver is the most used element in commercially available nanomaterial-containing products and Ag NPs were specifically mentioned in at least 424 product descriptions as of 2014 [2]. This widespread use of Ag NPs continues to raise concerns about how they might interact with biomolecules once they enter the physiological system. The interaction of NPs with biological cells follows various pathways, involving either penetration through the cell membrane followed by a direct attack on cell organelles [3]–[6], or by adhering to the cell membrane and, thereby, affecting the cell organelles indirectly [7], [8]. The former mechanism is possible when the particle size is <100 nm; such NPs can enter cells either actively or passively, depending on the cell type [6]. The latter possibility usually arises when the particle size is >100 nm.

For cells that are in proximity of silver nanoparticles there is a high probability of the NPs adhering to the cell membrane. It has been shown that the charge on the silver ion is crucial for its antimicrobial activity; the mechanism is based upon electrostatic attraction between the negatively-charged cell membrane of a micro-organism and the positive charge of the Ag^+^ comprising the Ag NPs [1]. Thiol-containing proteins in the cell membrane are likely to be one of the effective Ag^+^ ion binding sites [9]. The cysteine residues of NADH dehydrogenases that are present in NADH ubiquinonereductase complexes (Complex I) of E.coli may also be possible sites for Ag^+^ ion binding [10], [11]. One of the identified mechanisms by which Ag NPs act as antimicrobials is by binding to these sites.

The membrane of a red blood cell (RBC) contains proteins and glycoproteins embedded in a fluid lipid bilayer that exhibits viscoelastic behavior. Sialic acid rich glycoproteins of the RBC membrane are responsible for a negatively charged surface which creates a repulsive electric zeta potential between cells [12]. This electronegativity helps in preventing the interaction between RBCs and the other blood cells, and especially between each other [13]. The zeta potential is the electrostatic potential that exists at the shear plane of a particle; it is related to both surface charge and the particle's local environment. On the other hand, the electropositivity of Ag NPs makes them electrostatically attractive to RBC membranes. In general, the negatively charged cell membrane is known to show a tendency to adsorb positively charged or neutral nanoparticles [14]. The process of nanoparticles adsorption on cell membrane involves van der Waals forces, electrostatic charges, steric interactions and/or surface charges. The process of nanoparticle uptake by cells could be considered to comprise two steps: (*i*) the binding of nanoparticles to the cell surface and, (*ii*) the internalization of nanoparticles by specific endocytosis pathways [14]. RBCs are not expected to take up nanoparticles by endocytosis but their entry can be effected through the RBC membrane's ion transport channels. The possibility of nanoparticle internalization through cell membrane channels followed by their binding is limited and would be expected to be particle size dependent.

**Figure 1:**
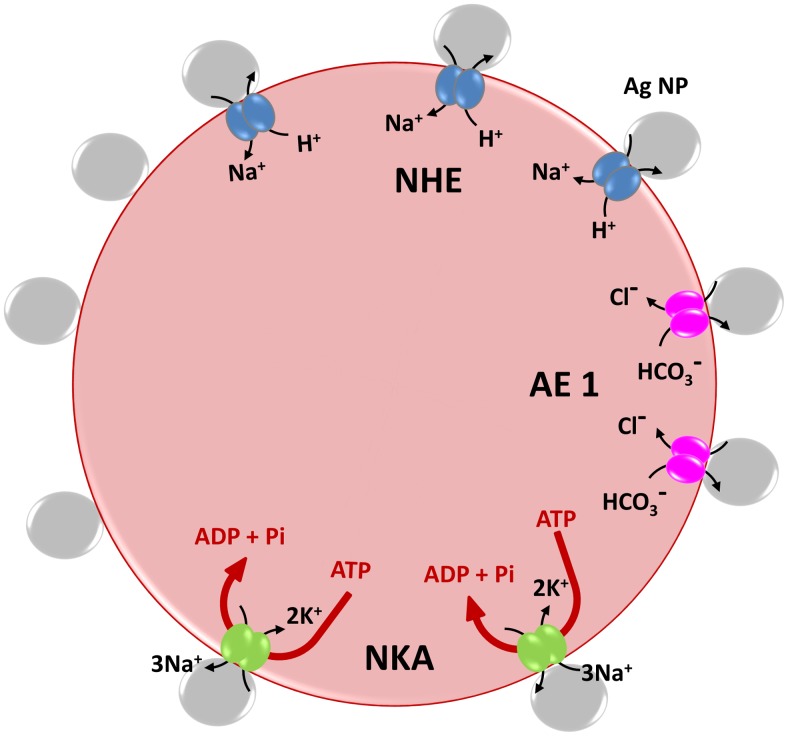
Schematic representation of the membrane channels in a red blood cell. The NPs are shown to be blocking the exchange channels in this cartoon representation.

The nature of the lipid bilayer associated with the RBC membrane is amphiphilic, like that of other eukaryotic cells: it is partially or totally permeable to gases (CO_2_, O_2_, N_2_) and small molecules (water, urea, ethanol) while being impermeable to charged polar molecules and ions (Na^+^, K^+^, Cl^−^, HCO_3_^−^) [15]. Polar molecules and ions are transported via facilitated diffusion, a process of passive transport aided by integral membrane proteins. The Cl^−^/HCO_3_^−^ exchange is carried out by the Anion Exchanger 1 (AE1) or Band 3 protein complex, the Na^+^/K^+^ exchange is carried out by sodium-potassium adenosine triphosphatase (NKA-Na^+^/K^+^-ATPase), an antiporter enzyme and Na^+^/H^+^ exchange is carried out by highly regulated (glyco) phosphoproteins known generically as Na^+^/H^+^ exchangers (NHEs) [15]. Figure 1 is a schematic representation of these channels on an RBC membrane. Improper functioning of these transport pathways may lead to changes in the chemical and mechanical properties of the cell membrane and even to pH imbalances, increase in ROS levels, or to conformational changes of hemoglobin (Hb) in the RBC cytoplasm. Ejection of intracellular H^+^ in exchange for external Na^+^ is the most effective means of eliminating excess acid from actively metabolizing cells [16]. This essential function is carried out by a family of antiporters, known generically as Na^+^/H^+^ exchangers (NHEs) [17]. Decrease in the intracellular pH (increase in acidity of intracellular environment) of RBCs will lead to the decrease in hemoglobin's oxygen binding affinity, as explained by the well-known Bohr Effect.

In this study we have investigated the possible effects of the adsorption/ adhesion of Ag nanoparticles (of average diameter 100 nm) on RBC membranes by monitoring Raman spectra with the emphasis on conformational changes in hemoglobin that occur on Ag NP treatment. These type of studies are important from the perspective of revealing both beneficial and adverse effects of nanoparticles in their interactions with living cells, especially in the context of a rapidly expanding nano-bio-technology market. A recent review has cogently listed several aspects of nanotechnological industrialization, exposure, and toxicity of Ag NPs [18]. Apart from their bactericidal activity, Ag nanoparticles exhibit toxicity towards mammalian cells. There are, however, ongoing debates on whether such toxicity is due to the nanoparticles themselves or to the silver ions (Ag^+^) they release [19], [20]. Our study focuses on intra-erythrocytic events that occur upon their exposure to silver nanoparticles whose dimensions are larger than the typical pore size in a human RBC membrane. The mechanism of nanoparticle cytotoxicity we discuss in the context of our findings with human erythrocytes may also be applicable to any other non-phagocytic cell.

## Materials and Methods

Intravenous blood was drawn from healthy, informed volunteers through venipuncture in an EDTA coated vacutainer after obtaining written, informed consent. Ethical approval for the project on “Development of Raman Tweezers for studies on human red blood cells” and, explicitly to draw blood from informed volunteers for this project, was obtained from the University Ethics Committee, Manipal University, on 11 December 2008 (Ref. UEC/35/2008) and renewed on 14th January 2012 (Ref. UEC/Renewal-07/2012). The non-coagulated whole blood was centrifuged at 3000 rpm for 5 minutes to discard the plasma. 100 µl of this blood was diluted in 4 ml of complete RPMI 1640 (RPMI+10% BSA) medium. The RBC suspension was treated with 1 µl, 5 µl and 10 µl of AgNO_3_ nanoparticles. The AgNO_3_ nanoparticles used in this study were commercially procured (Sigma-Aldrich, India) with specified average particle size of ∼100 nm. The control RBC suspension and NP-treated suspension (maintained in different culture wells) were incubated at 37°C with 5% CO_2_ for 45 hours. Both the incubated samples were further diluted with phosphate buffered solution (PBS) for Raman Tweezers spectroscopy study. The Raman spectroscopy was performed 20 hours and 45 hours after incubation.

Micro-Raman spectroscopy of incubated control and Ag NP-treated RBCs was performed using a home-built, single-beam Raman Tweezers set-up. Our set-up uses a tightly-focused 785 nm wavelength laser beam (from a Starbright Diode Laser) to simultaneously trap and excite live RBCs suspended in a biological medium. The tight focusing of our laser beam is achieved using a high numerical aperture (1.3 NA), 100X oil immersion microscope objective of a Nikon Eclipse Ti-U microscope with supportive optics. The scattered Raman signals are directed towards a Horiba JobinYvon Spectrometer equipped with a 1200 gr/mm holographic grating. A Symphony CCD detector is utilized to collect the Raman spectra of trapped cells. The complete system design, specifications, and performance have been discussed elsewhere [21], [22].

The Raman shifts recorded in the present series of experiments were in the 450–1800 cm^−1^ range. Typical resolution was 5.7 cm^−1^ and the accuracy with which spectral lines were identified was ±3 cm^−1^ (0.2 nm).

## Results and Discussion

**Figure 2:**
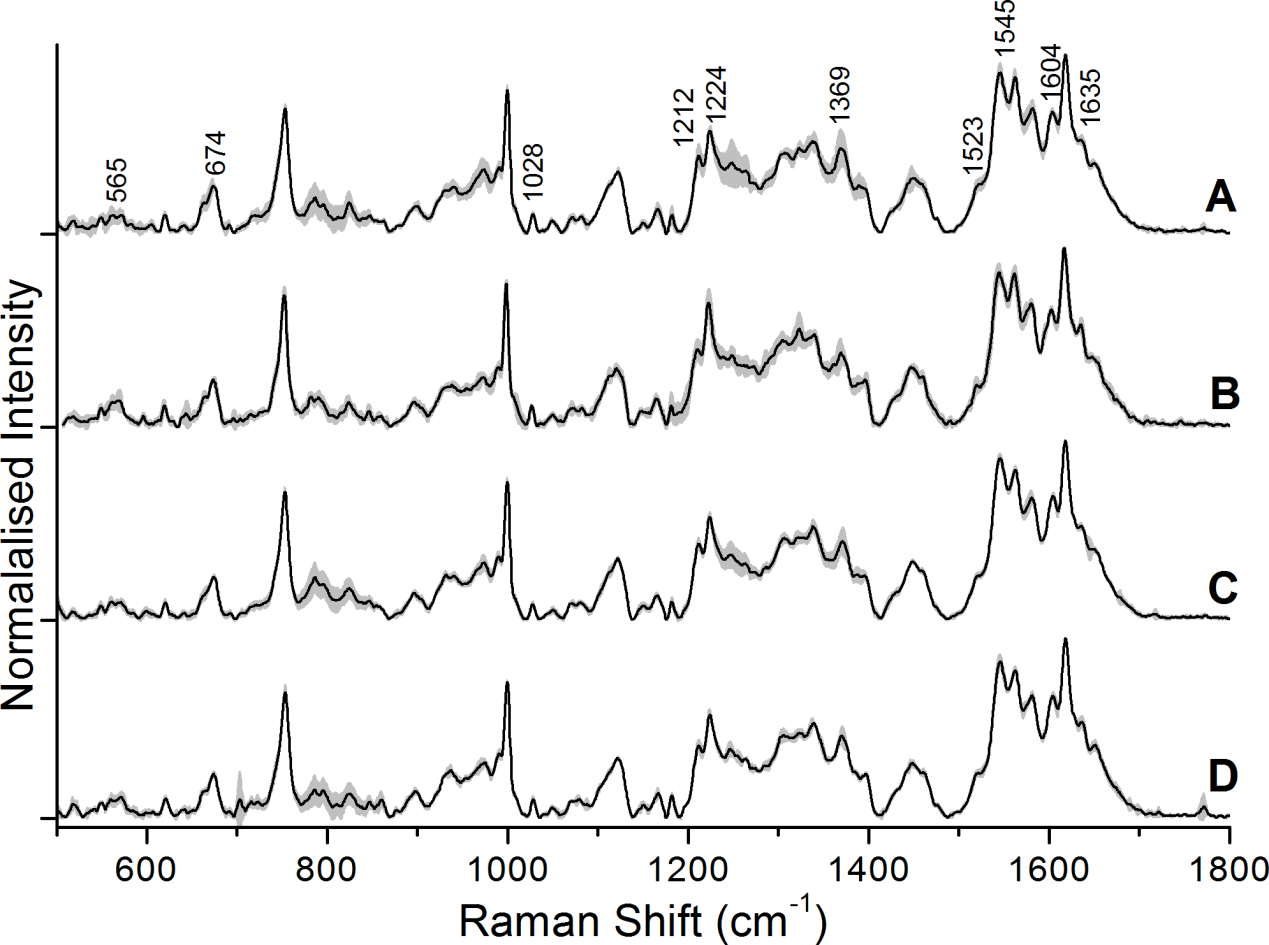
The average spectra of control and Ag NP treated RBCs 20-incubation: (A) Control, (B) with 5 µl Ag NPs, and (C) with 10 µl Ag NPs.

**Figure 3:**
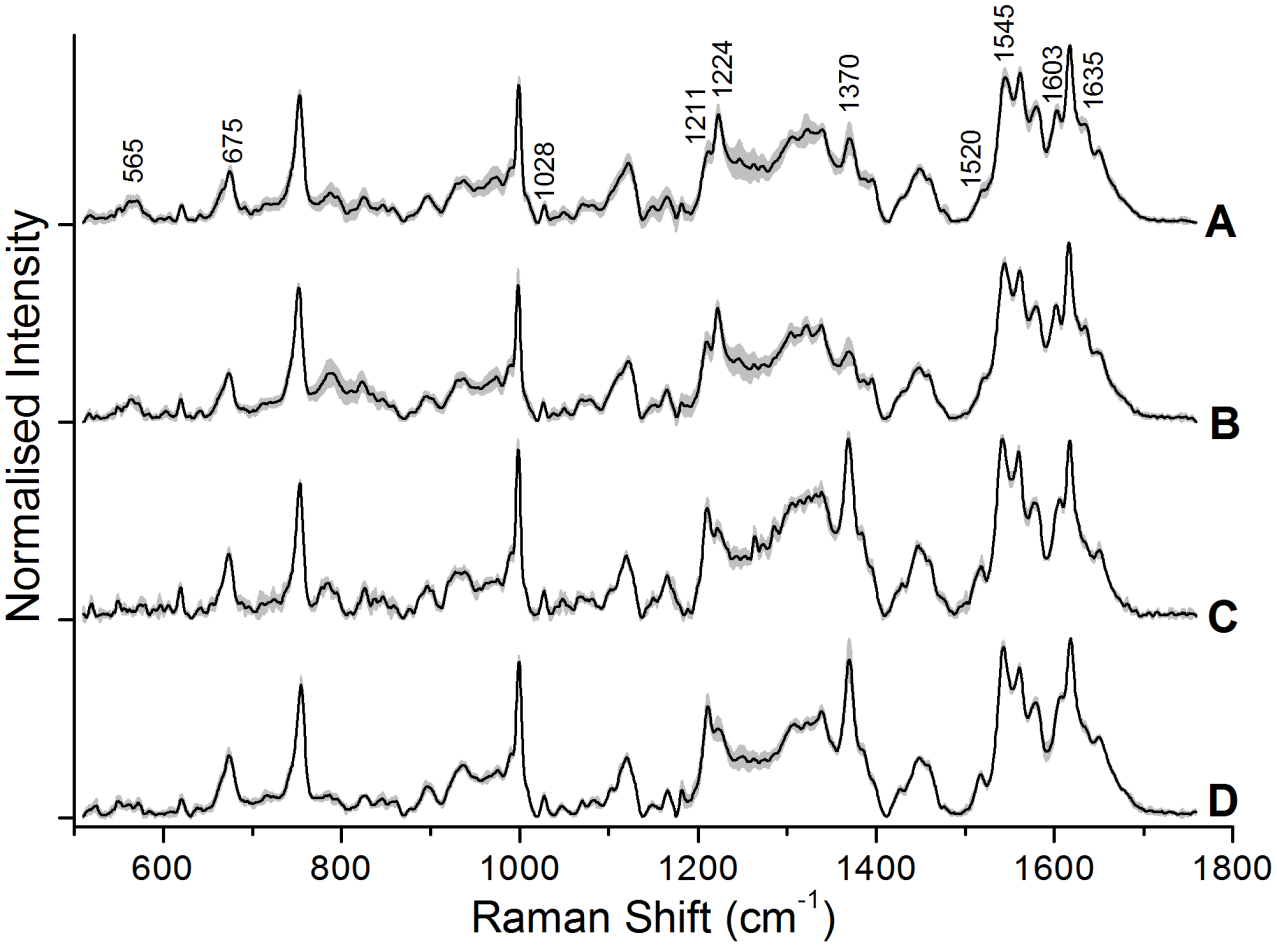
The average spectra of control and Ag NP treated RBCs 45-incubation. (A) Control, (B) 1 µl Ag NPs, (C) 5 µl Ag NPs, and (D) 10 µl Ag NPs.

Figure 2 and Figure 3 show typical (average) Raman spectra from Control and Ag NP treated RBCs recorded after 20 hours and 45 hours of incubation, respectively. We carried out frequency assignments of the recorded spectra based on the notation system used by Abe *et al.* [23] and Hu *et al*. [24], with some additional assignments from Parker [25]. Detailed Raman frequency assignments of a typical Raman spectrum from erythrocytes recorded by our set-up have been listed elsewhere recently [21]. Raman tweezers spectroscopy performed after 20 hours of incubation showed no significant spectral alterations between control RBCs and Ag NP-treated RBCs. On the other hand, Raman tweezers spectroscopy performed after an incubation period of 45 hours did show significant changes in the Raman spectra of 5 µl and 10 µl Ag NP treated RBCs compared to those of control RBCs, whereas 1 µl treated RBC spectra showed no difference.

In Figure 2, the Control spectrum is an average of 5 spectra, each measured from 5 control RBCs; the 1 µl Ag NP spectrum is an average of 4 spectra from four Ag NP (1 µl) treated RBCs; the 5 µl Ag NP spectrum is an average of 10 spectra from 10 Ag NP (5 µl) treated RBCs; and the 10 µl Ag NP spectrum is an average of 5 spectra from 5 Ag NP (10 µl) treated RBCs. In Figure 3, the Control and 1 µl Ag NP spectra are an average of 15 spectra, each measured from 15 control RBCs and 15 Ag NP (1 µl) treated RBCs, whereas the 5 µl Ag NP spectrum is an average of 5 spectra from 5 Ag NP (5 µl) treated RBCs; the 10 µl Ag NP spectrum is an average of 8 spectra from 8 Ag NP (10 µl) treated RBCs.

The major changes that we observe between the average spectra of control and Ag NP (5 µl and 10 µl) treated RBCs are in the vibrations related to Fe-O_2_ stretching [ν(Fe-O_2_)] at 565 cm^−1^, pyrrole half-ring stretching [ν(pyr half-ring)_sym_] at 1370 cm^−1^, pyrrole deformation mode [*δ*(pyr deform)_sym_] at 675 cm^−1^, venyl deformation mode [*δ*( = C_b_H_2_)_4_] at 1028 cm^−1^, the asymmetric stretching mode [ν(C_α_C_m_)_asym_] at 1635 cm^−1^, the skeletal stretching modes [ν(C_β_C_β_)] at 1520 cm^−1^, 1545 cm^−1^ and 1603 cm^−1^ and finally, the methine C-H deformations [*δ*(C_m_H)] at 1211 cm^−1^ and 1224 cm^−1^.

**Figure 4:**
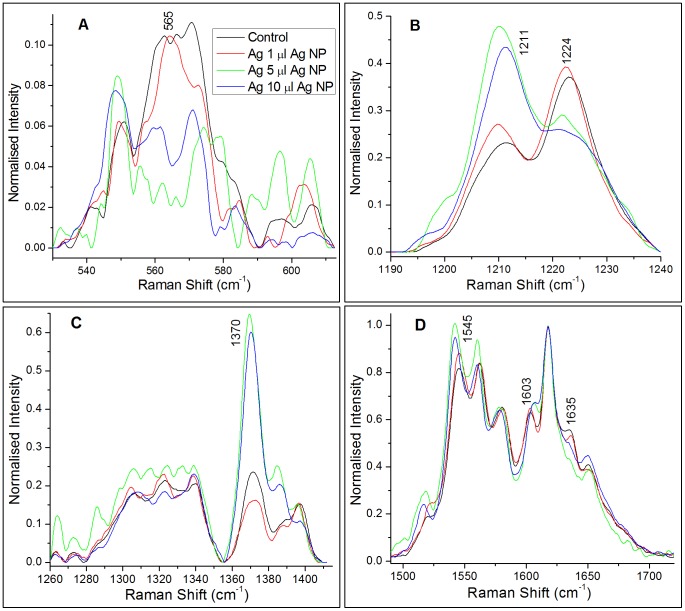
Interpolated plot of control and Ag NP treated RBCs 45-incubation in the A) Fe-O_2_ stretching region, B) methine C-H deformation region, C) pyrrole half ring symmetric stretching region, and D) skeletal mode region.

We observe that the intensity of the Raman peak at 1211 cm^−1^ increases whereas that at 1224 cm^−1^ decreases in the spectra of 5 µl and 10 µl Ag NP treated RBCs compared to those in control RBC spectra. Both the peaks concurrently originate from the methine ( = C_m_H−) C-H deformation (ν_13_) in hemes and are sensitive to the deformation angle of the methine group. Figure 4B shows the overlaid plot of the average spectra from control and Ag NP treated RBCs in the methine C-H deformation region. A conformational change of hemoglobin brings methine C-H vibrations close to the protein subunits and this influences a change in the deformation angle of C-H vibrations [26]. As a result the C-H vibrational frequency in that heme molecule shifts from 1224 cm^−1^ to 1211 cm^−1^. In the Raman spectra of an RBC, this is reflected by a decrease in intensity of the 1224 cm^−1^ peak and concurrent increase in that of the 1211 cm^−1^ peak. Wood *et al*. [26] have suggested that such frequency shifts represent conformational changes in heme that occur during its transition from the deoxygenated state to the oxygenated state. Hence, inside the nanoparticle treated RBCs most of the Hb molecules are permanently modified to the T-state whereas inside control RBCs more of the Hb molecules exist in the R-state. It is evident from our observations that the nanoparticles are blocking the membrane transport channels and thereby, reducing the oxygen binding affinity of hemoglobin through increase in H^+^ concentration. In the T state of hemoglobin the C-terminal histidine (His HC3) of the β-subunits participate in the formation of ion pairs with Asp FG1 [27] and the proximity to Asp FG1 gives His HC3 a higher pKa than normal, so it is protonated. In other words, the lower pH (higher H^+^) stabilizes the T-state of hemoglobin. Ion pairs that stabilize the T state of deoxy-hemoglobin must break to form the R state, which is not expected to happen at lower intracellular pH. There are many reports on nanomaterials-cell interaction studies where nanoparticles act as membrane channel blockers [28], [29]. Bhabra *et al.* [30] reported that cobalt–chromium nanoparticles induced DNA damage in human fibroblast cells without physically crossing the cell membrane. These studies support our hypothesis on the indirect effect of Ag NPs on hemoglobin stability.

Significant changes have been observed in the well-known spin-state marker region (1500–1650 cm^−1^) of heme. The increase in intensity of other T-state marker peaks located at 1520 cm^−1^, 1545 cm^−1^, and 1603 cm^−1^, corresponding to the C_β_C_β_ skeletal stretching modes in the spectra of 5 µl and 10 µl Ag NP treated RBCs, indicates the presence of more deoxy hemes in these samples [26], [31]. Furthermore, the decrease in intensity of the R-state marker peak at 1635 cm^−1^, corresponding to ν(C_α_C_m_)_asym_ in the spectra of 5 µl and 10 µl Ag NP treated RBCs, is an indication of the formation of deoxy hemoglobin, as suggested earlier [26], [31]. Figure 4D shows the overlaid plots of average spectra from control and Ag NP treated RBCs in the spin-state marker region.

It is also clear from Fig. 4D that the 1520 cm^−1^ and 1545 cm^−1^ peaks show a frequency downshift of ∼3 cm^−1^ while the peak at 1603 cm^−1^ shows a frequency upshift of ∼4 cm^−1^ in the spectra of NP-treated RBCs. This decrease/increase in frequency of skeletal C-C stretching may be influenced by the pull of pyrrole –N terminal by Fe.

Another noteworthy feature of our study is the dramatic decrease in intensity of the well-established marker peak for heme bound O_2_, the Fe-O_2_ stretching mode (565 cm^−1^) in 5 µl and 10 µl Ag NP-treated RBCs spectra. This confirms our view that Ag NP induced distortions in the RBC membrane have permanently transformed most of the hemoglobin molecules in it into the deoxy state [22]. Figure 4A shows the overlaid plots of the average spectra from control and Ag NP-treated RBCs in the low frequency region. As expected, the Fe-O_2_ peak is also prominent in the average spectra of Control and Ag-treated RBCs recorded after 20 hours of incubation (see Fig. 2), indicating that the Ag NPs have not affected the hemoglobin in this time frame.

On the other hand, the Raman peaks from heme, located at 675 cm^−1^ - which correspond to pyrrole deformation - and at 1370 cm^−1^ - which correspond to pyrrole half-ring symmetric bending - show intensity enhancement in the spectra of 5 µl and 10 µl Ag NP-treated RBCs. Figure 4C shows the overlaid plot of the average spectra from control and Ag NP treated RBCs in the pyrrole half-ring symmetric bending region. The increase in intensity of these peaks can be attributed to the increased exposure of contributing bonds to the excitation photons. In the T (deoxy) state of hemoglobin, the Fe-ion is pulled out of the heme plane toward the histidine residue to which it is attached and, as a result, the heme is exposed out of the protein subunits. Hence in the T state, the pyrrole skeleton is more exposed to the photons compared to that in the R state.

The 785 nm excited Raman spectra of RBCs are also rich in the vibrations that originate from protein subunits of hemoglobin. The relative intensities of the peaks at 999 cm^−1^, 1449 cm^−1^ and 1650 cm^−1^, attributed to phenylalanine, CH_2_/CH_3_ deformation in amino acid side chains and amide I, respectively, show some difference between spectra of control and Ag NP-treated RBCs.

The enhancement in the intensity of specific vibrations in deoxyhemoglobin with 785 nm excitation is a matter of concern. Eaton *et al.* [32] reported both experimental and theoretical analyses of optical spectra of heme moieties in oxygenated and deoxygenated hemoglobin and observed that deoxyhemoglobin exhibits a weak charge transfer band III around 760 nm whereas oxyhemoglobin displays a broad band centered at 925 nm. The extinction coefficients of oxyhemoglobin and deoxyhemoglobin have a isosbestic point at 805 nm and this is the basis for near-infrared spectroscopy (NIRS) applications in monitoring the changes in oxygenated and deoxygenated hemoglobin concentration in organs like the brain [33]–[36]. The work reported by Wood *et al.* [26] supported the intensity enhancements observed in oxyhemoglobin and deoxyhemoglobin excited at 785 nm by considering the effects of vibronic coupling of charge transfer band III and band IV with Soret band through a low-frequency vibrational mode of B_1g_ symmetry. The enhancement phenomena through the coupling of band III assigned to 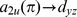 transition by Eaton *et al.* [32] and the Soret band was rationalized by Franzen *et al.* [37]. A thorough analysis of mode specific resonance enhancements in functional hemoglobin of a live RBC has to be performed to understand the underlying mechanism for the drastic change in vibrational peak intensities of oxy- and deoxy-hemoglobin. The data gathered from such studies will help one to deduce the related conformational changes that occur during oxygenation and deoxygenation of hemoglobin in functional erythrocytes and related disorders.

**Figure 5:**
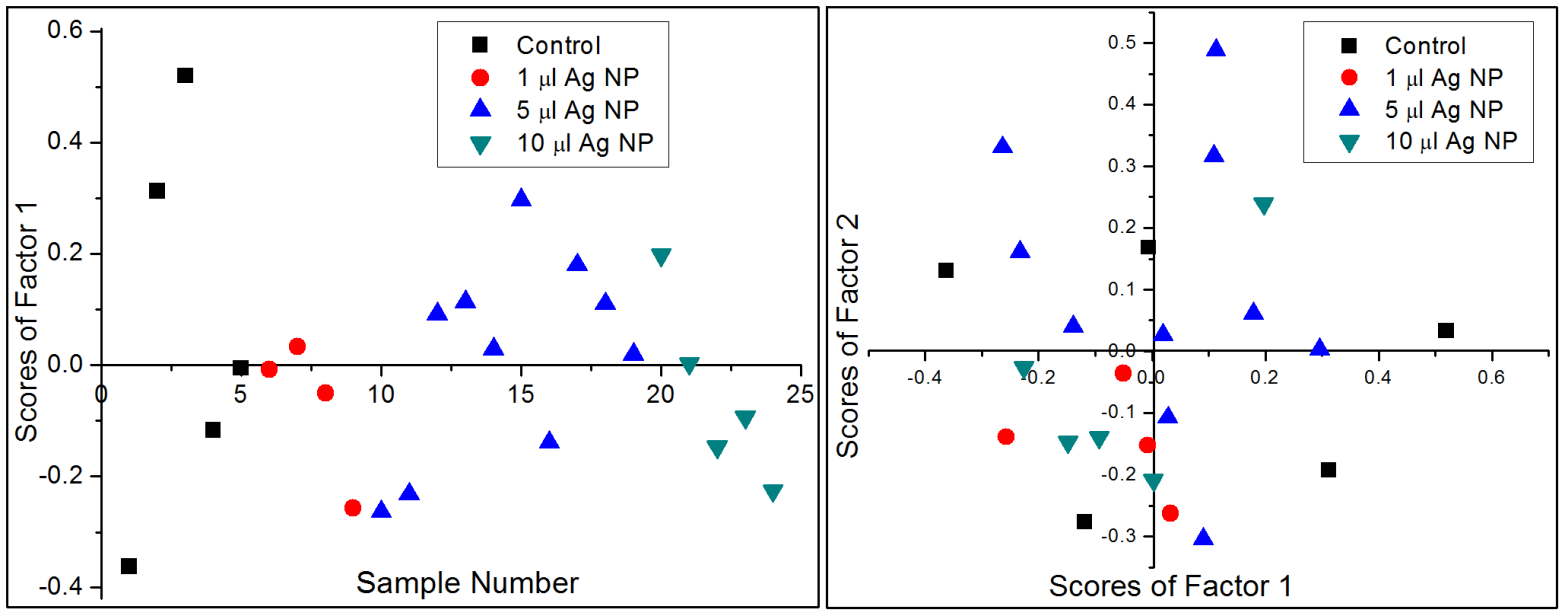
PCA results for Raman spectra of control and Ag NP treated RBCs after 20 hours of incubation. The left panel shows sample number versus scores for PCA factor 1 and the right panel depicts scores of factor 1 versus scores of factor 2.

**Figure 6:**
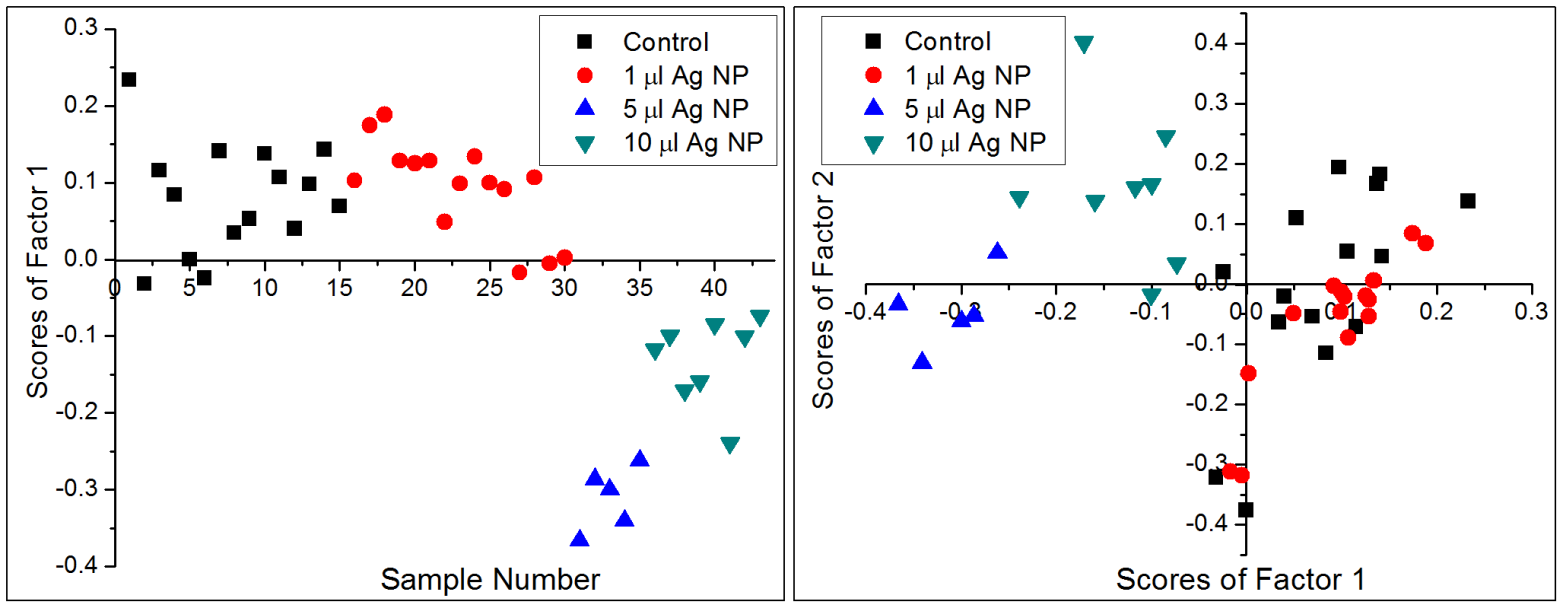
PCA results for Raman spectra of control and Ag NP treated RBCs after 45 hours of incubation. The left panel shows sample number versus scores for PCA factor 1 and the right panel depicts scores of factor 1 versus scores of factor 2.

We carried out principal component analysis (PCA) to statistically differentiate the micro-Raman spectra of control and Ag NP treated cells 20 hours and 45 hours post-incubation. PCA was performed separately on a total of 24 spectra recorded 20 hours post-incubation and on 43 spectra recorded 45 hours post-incubation. Figures 5, 6 show our PCA results for 20 hours post-incubation and 45 hours post-incubation, respectively. It is clear from Fig. 5 that there is no clear discrimination between the Raman spectra of control and Ag NP treated RBCs: in both the left and right panels the data is seen to be spread out and interspersed. This is also evident from Fig. 2, where the average spectra do not show any changes. On the other hand Fig. 6 (45 hours post-incubation) shows a clear difference between two classes: control and 1 µl as one class and 5 µl and 10 µl as another class. In this case we note that the first factor itself defines the data well, with more than 62% variance among the spectra. The left panel in Fig. 6 shows a plot of sample number versus scores of factor 1. It is clear from the plot that most of the control and 1 µl Ag NP treated spectra have positive scores and all the spectra of cells treated with 5 µl Ag NP and 10 µl Ag NP have negative scores. The right panel of Fig. 6 shows the plot between scores of first factor and the scores of second factor. This plot clearly shows that most of the control and 1 µl Ag NP treated samples lie in the first and fourth quadrants while almost all 5 µl Ag NP and 10 µl Ag NP samples lie in the second and third quadrants. These results constitute clear supporting evidence for the peak intensity variations that we presented in Figs. 3, 4.

**Figure 7:**
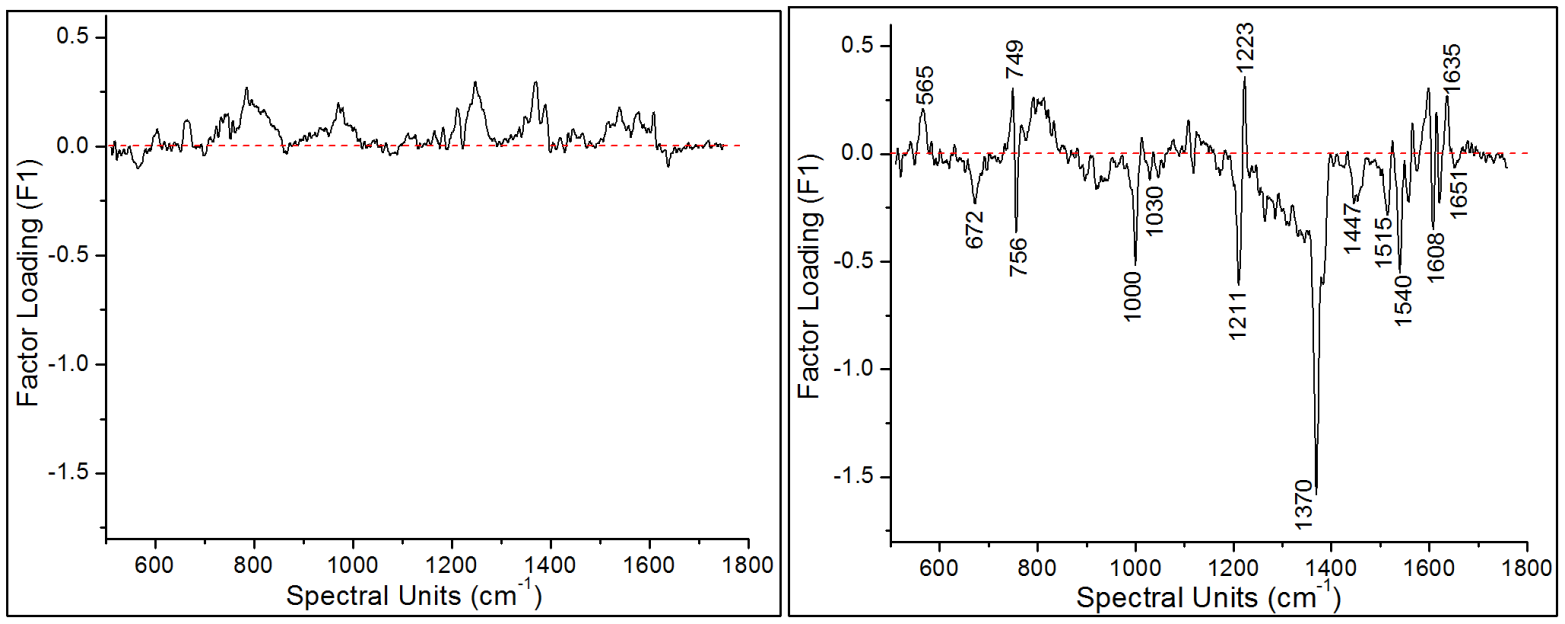
Plot of PC1 (F1) loadings for control and Ag NP-treated RBC spectra after 20 hour (left) and 45 hours (right) of incubation.

The PC 1 (F1) loadings plots presented in Fig. 7 enable some insight into the spectral changes that characterize indirect effect of Ag NPs on the conformation and dynamics of hemoglobin in functional erythrocytes. The right panel of Fig. 7 (after 45 hours of incubation) shows a clear discrimination between the Raman peaks associated with the oxygenated state of hemoglobin (565 cm^−1^, 1223 cm^−1^ and 1635 cm^−1^) in control RBCs and the deoxygenated state (672 cm^−1^, 1211 cm^−1^, 1370 cm^−1^, 1515 cm^−1^, 1540 cm^−1^ and 1608 cm^−1^) of hemoglobin in Ag NP (5 µl and 10 µl) treated RBCs. On the other hand, the left panel of Fig. 7 (after 20 hours of incubation) indicates no visible difference among two groups of the spectra except for small cell-to-cell variations. The PC1 loadings plot for 45 hours post incubation also shows the splitting of the band corresponding to the B_1g_ mode at 753 cm^−1^ into two peaks: at 749 cm^−1^ for pyrrole deformation (ν_16_) and at 756 cm^−1^ for pyrrole breathing (ν_15_).

**Figure 8:**
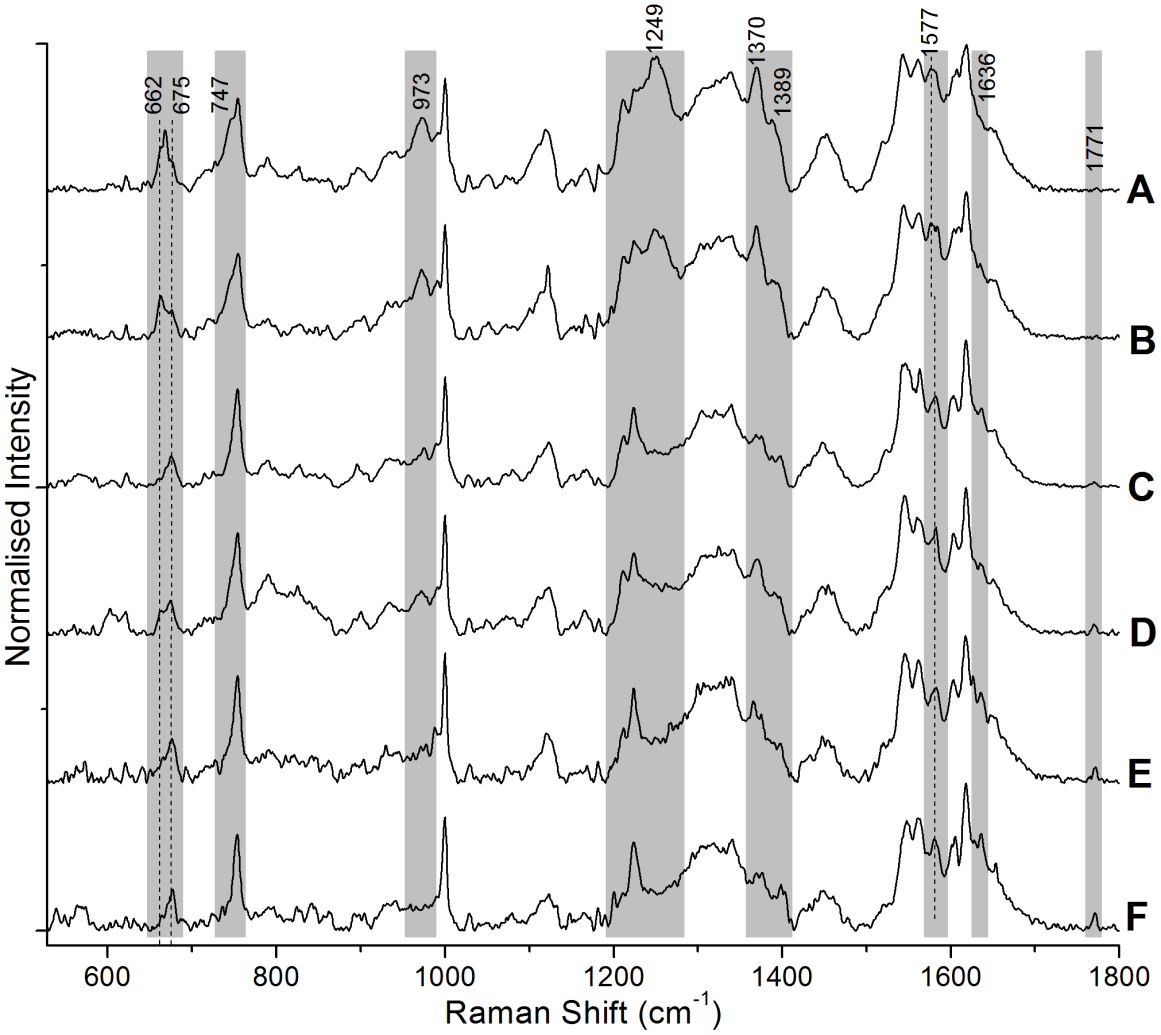
Power dependent Raman spectra of single, live RBCs. The incident power levels were A) 20 mW, B) 15 mW, C) 12 mW, D) 10 mW, E) 5 mW, and F) 4 mW.

We also recorded the Raman spectra of RBC at different trapping/excitation powers (at 785 nm) to check the power dependent variations in the spectrum. All the 6 spectra shown in Fig. 8 were recorded from 6 different RBCs that were trapped and Raman excited at different laser powers. In all our studies, the present experiments as well as those reported earlier [21], [22], [38]–[40], the powers levels mentioned by us are those at the sample, after microscope objective (100X), measured using an integrating sphere. It is clear from Fig. 8 that significant changes in the spectra are only observed at excitation power levels in excess of 15 mW: there are no obvious changes till 12 mW of power, as shown in Fig. 8 (C–F). The Raman spectra at 15 mW and 20 mW of power show a series of changes in the spectral features: a total of 4 new peaks appear at 662 cm^−1^, 1249 cm^−1^, 1389 cm^−1^ and 1577 cm^−1^ while peaks at 973 cm^−1^ and 1370 cm^−1^, which were already present in the low power spectra, show dramatic increase in intensity. The peaks at 662 cm^−1^, 973 cm^−1^, 1249 cm^−1^, 1370 cm^−1^, and 1389 cm^−1^ are identified as heme aggregation markers [41]. Apart from these changes, the venyl mode (ν_10_) at 1636 cm^−1^ and the amide I band at 1771 cm^−1^ both show the expected power-dependent decrease in intensity. The amide I band is almost missing in the Raman spectra at 15 mW and 20 mW excitation powers, suggesting photo-induced dissociation of polypeptide bonds in proteins. The better resolution spectra recorded in the present experiments establish that, even at high power, the intensity of the 1211 cm^−1^ peak is less than that of the 1224 cm^−1^ peak, and that the appearance of the 1249 cm^−1^ peak does not mask the 1224 cm^−1^ peak.

The 662 cm^−1^ peak appears as a shoulder to the 674 cm^−1^ peak corresponding to pyrrole deformation. At 20 mW, a peak at 668 cm^−1^ also accompanies the 662 cm^−1^ peak. The pyrrole deformation (ν_16_) peak appears at 747 cm^−1^ as a shoulder to the 753 cm^−1^ peak and the 1577 cm^−1^ peak appears as a shoulder peak to the already present 1583 cm^−1^ peak of ν_37_ mode.

Ahlawat *et al.* [42] studied the 1064 nm laser beam induced changes in the RBC spectra and observed increase in the intensity of 1547 cm^−1^ and 1212 cm^−1^ along with a concomitant decrease in intensity of peaks at 1224 cm^−1^ and 1620 cm^−1^ which are reversible up to the trapping power of 20 mW at 1064 nm. As we know that the photo-induced cell damage is wavelength dependent and decreases as we move towards the infrared region, the damage threshold is more in the case of the 1064 nm laser beam.

On the basis of our power-dependent measurements it is possible to be certain that the spectral changes observed in the Ag NP-treated RBCs, including the intensity flipping of the peaks at 1211 cm^−1^ and 1224 cm^−1^, are not photo-induced. The power levels that are used to record the Raman spectra of control and Ag NP-treated cells are well below the threshold for occurrence of photo-dissociation and/or heme aggregation. Hence, the spectral changes presented by us in Figs. 3, 4 are entirely ascribable to Ag NP-induced changes in the heme conformations.

## Summary

The information collected by us using Raman tweezers spectroscopy on single, live erythrocytes exposed to the influence of silver nanoparticles (of diameter >100 nm) has revealed that Ag NPs induce irreversible modifications in the heme's conformation through a transformation from the R-state (oxy state) to the T-state (deoxy state). We rationalize our observations by proposing a model for the nanoparticle cytotoxicity pathway when NP's size is larger than the membrane pore size. Specifically, we propose that the Ag NPs interact with the cell surface in such manner that induces damage to the heme molecule in the RBC due to the alteration of intracellular pH caused by the blockage of cell membrane transport.
